# Neural correlates of visual hallucinations in dementia with Lewy bodies

**DOI:** 10.1186/s13195-014-0091-0

**Published:** 2015-02-17

**Authors:** Camille Heitz, Vincent Noblet, Benjamin Cretin, Nathalie Philippi, Laurent Kremer, Mélanie Stackfleth, Fabrice Hubele, Jean Paul Armspach, Izzie Namer, Frédéric Blanc

**Affiliations:** Neuropsychology Unit, Neurology Department, University Hospital of Strasbourg, 1, avenue Molière, 67098 Strasbourg, France; CMRR (Memory Resource and Research Center), University Hospital of Strasbourg, 1, avenue Molière, 67098 Strasbourg, France; University of Strasbourg and CNRS, ICube Laboratory UMR 7357, IMIS team and FMTS (Strasbourg Federation of Translational Medicine), 1, place de l’Hôpital, 67000 Strasbourg, France; Department of Nuclear Medicine, University Hospital of Strasbourg, 1, avenue Molière, 67000 Strasbourg, France

## Abstract

**Introduction:**

The aim of this study was to investigate the association between visual hallucinations in dementia with Lewy bodies (DLB) and brain perfusion using single-photon emission computed tomography.

**Methods:**

We retrospectively included 66 patients with DLB, 36 of whom were having visual hallucinations (DLB-hallu) and 30 of whom were not (DLB-c). We assessed visual hallucination severity on a 3-point scale of increasing severity: illusions, simple visual hallucinations and complex visual hallucinations. We performed voxel-level comparisons between the two groups and assessed correlations between perfusion and visual hallucinations severity.

**Results:**

We found a significant decrease in perfusion in the left anterior cingulate cortex, the left orbitofrontal cortex and the left cuneus in the DLB-hallu group compared with the DLB-c group. We also found a significant correlation between decreased bilateral anterior cingulate cortex, left orbitofrontal cortex, right parahippocampal gyrus, right inferior temporal cortex and left cuneus perfusion with the severity of hallucinations.

**Conclusions:**

Visual hallucinations seem to be associated with the impairment of anterior and posterior regions (secondary visual areas, orbitofrontal cortex and anterior cingulate cortex) involved in a top-down and bottom-up mechanism, respectively. Furthermore, involvement of the bilateral anterior cingulate cortex and right parahippocampal gyrus seems to lead to more complex hallucinations.

## Introduction

In people older than 65 years of age, dementia with Lewy bodies (DLB) is the second most common cause of neurodegenerative dementia after Alzheimer disease (AD) [1,2]. Visual hallucinations (VHs) are one of the commonest features of DLB, present in 54% to 70% of patients [3]. It most frequently consists of simple visual illusions wherein objects are distorted or deformed, even though more complex hallucinations may also occur.

Different hypotheses have been proposed to explain the occurrence of VHs in DLB. One of the main hypotheses is that VHs could be consecutive to visuospatial, visuoperceptual or attention deficits, which are more frequent in DLB with VHs than in either DLB without VHs or AD [4]. These visual deficits of central origin are coherent with the specific occipital hypoperfusion in DLB [5]. Another hypothesis is a dysregulation of the gating and filtering of external perception and internal image production [6]. The developers of the Perception and Attention Deficit model proposed that VHs are caused by a combination of impaired attentional binding (top-down) and perceptual processes (bottom-up) [7].

To support theses hypotheses, some study researchers have investigated the relationship between VH and cerebral perfusion or metabolism [8-15] or atrophy by using magnetic resonance imaging (MRI) [16] in patients with neurodegenerative disease. Howard *et al*. [8] found a decreased reaction of the visual cortex to a visual stimulus during hallucinations in a patient with DLB. Nagahama *et al*. [10] studied 100 patients with DLB patients with or without hallucinations using single-photon emission computed tomography (SPECT). Those authors demonstrated the involvement of both posterior (bilateral occipital and parietal cortices) and less significantly anterior regions (bilateral middle frontal gyri and bilateral posterior cingulate gyri) in the group with hallucinations. In another study, Pernezcky *et al*. [13] suggested the involvement of hypometabolism in both visual associative areas (right temporo-occipital cortex) and the prefrontal region (right middle frontal gyrus). Moreover, the involvement of the anterior region was demonstrated in a volumetric study in which Sanchez-Castaneda *et al*. [16] showed right inferior frontal gyrus atrophy in patients with DLB with VHs. The authors supposed that the prefrontal region is involved in insight into and consciousness of the hallucinations.

The aim of our study was to investigate the neural basis of VHs in DLB and, moreover, the qualitative intensity of VH, which has never previously been investigated in DLB, to the best of our knowledge. Working with the hypothesis that a deficit both in visual treatment of information and in executive control could contribute to VH, we posited that the group of DLB patients with VH would have greater hypoperfusion in posterior regions (that is, occipital and parietotemporal cortices) and the anterior region (that is, prefrontal cortex), respectively, as compared with the group without VH.

## Methods

### Ethics statement

Our study did not need ethical approval or the patients’ written consent according to French legislation, because it was a retrospective study and SPECT was performed during the patients’ follow-up.

### Methodology

We conducted a retrospective study of patients diagnosed with DLB by three neurologist experts in dementia in the Memory Clinic of the Department of Neurology, University Hospital of Strasbourg, France, between 2006 and 2010. To be included, the patients had to have a probable or possible DLB diagnosis as defined by McKeith’s 2005 criteria [17], and a SPECT scan had to have been performed during the patients’ follow-up. SPECT is included in routine diagnostic workups of these patients and is performed for all patients to help in making the diagnosis. To distinguish DLB from Parkinson disease associated with dementia, we excluded patients in whom cognitive impairment had occurred more than 2 years after they were diagnosed with the extrapyramidal syndrome.

### Inclusion and exclusion criteria

We studied 100 patients’ records, and a total of 66 patients were included in the study. Nineteen patients were excluded because they had not undergone a SPECT scan; twelve patients were excluded because the diagnosis was uncertain (AD for two patients, trisomy 21 for three patients, epilepsy with memory deficit for two patients, vascular dementia for two patients, metabolic encephalopathy for one patient, Parkinson disease for one patient and no diagnosis for one patient); two patients were excluded because they had only auditory hallucinations; and one patient was excluded because he did not speak French. We also excluded patients with clinical features that could be explained by another cause, patients whose clinical records were incomplete and patients with hallucinations of a nonvisual type (for example, auditory, somatosensory). However, we included patients with another type of hallucination (*n* = 6) if they were associated with VHs.

### Patient records

Patients’ records were analyzed for the following items: sex, age, family history, personal history of depression, presence and severity of an extrapyramidal syndrome (tremor, extrapyramidal rigidity or akinesia), existence and type of hallucinations, presence of motor or cognitive fluctuations, Mini Mental State Examination (MMSE) and neuropsychological assessment, presence of a psychiatric disease or a sleep disorder evocative of a rapid eye movement sleep disorder, results of other investigations (cerebrospinal fluid biomarkers, including tau, phosphorylated tau, Aβ_1–42_ (Innogenetics, Ghent, Belgium), brain [^123^I]FP-CIT SPECT, brain MRI, electroencephalography) and patients’ medications at the time of the SPECT. By using the Unified Parkinson’s Disease Rating Scale III score [18], akinesia, rigidity and tremor were rated from 0 to 4 (0 = no symptoms to 4 = serious impairment).

### Assessments

Hallucinations were assessed by neurology experts. The patients were asked the following question: “Have you ever seen things that do not exist?” Different types of tests were applied in different patients during follow-up to evaluate cognitive function. The most frequently used tests were the Free and Cued Selective Reminding Test (FCSRT) [19] for episodic memory, the Frontal Assessment Battery (FAB) [20], Trail Making Test (TMT) A and B [21] and formal and semantic lexical evocation [22] for executive function; and a digit-span test for attention and working memory and the Rey-Osterrieth Complex Figure Test [23] for visuoconstructive function.

### Single-photon emission computed tomography

#### Image acquisition

A SPECT scan was obtained by a nuclear medicine physician for every patient during follow-up. The procedure used was as follows. The patients received an injection of 740 MBq of [^99m^Tc]ethyl cysteinate dimer (Neurolite; Lantheus Medical Imaging, North Billerica, MA, USA) (eight patients received 740 MBq of [^99m^Tc]exametazime Ceretec; GE Healthcare, Little Chalfont, UK). The image acquisition began 15 minutes after injection with a dual-head gamma camera (Siemens Medical Imaging, Hoffman Estates, IL, USA) equipped with a fan beam collimator specially manufactured for the study of the brain. Patients were imaged while in the supine position. The heads of the gamma camera were 15 cm away from the center of rotation. The height of the table was 20 cm. Image acquisition included 32 tomographic projections of 50 seconds each. The acquisition matrix was 128 × 128 pixels with zoom set at 1.23. The acquisition window was focused on the energy of the ^99m^Tc isotope photopeak (that is, 140 keV) with a window width of 15%.

#### Image processing

For image processing, we used SPM8 software (Statistical Parametric Mapping; Wellcome Department of Imaging Neuroscience, University College London [24]) running on MATLAB R2010a (MathWorks, Natick, MA, USA). SPECT images of each patient were spatially normalized to the Montreal Neurological Institute space. Intensities were linearly scaled using the average perfusion of the central regions of cerebellum because these areas are almost preserved in patients with DLB. Finally, images were smoothed with a Gaussian kernel of 12 mm.

### Statistical analysis

Patients were divided into two groups: a group of patients with DLB who were having VHs (DLB-hallu) and a control group of patients with DLB who were not having VHs (DLB-c). There were 36 patients in the DLB-hallu group and 30 patients in the DLB-c group.

We used the voxel-based statistical framework provided in SPM8 to compare the images of the two groups. We performed voxel-level comparison of perfusion of the DLB-hallu group with that of the DLB-c group using a two-sample *t*-test with age and type of tracer as covariates. Statistical maps were thresholded with *P* < 0.001 with a minimum cluster size of 25 voxels.

A second one-tailed analysis was then undertaken to investigate the putative correlation between perfusion intensity and the severity score of hallucinations while still considering age and type of radiotracer as covariates. We also chose a threshold of *P* < 0.001 and a minimum cluster size of 25 voxels.

These analyses were conducted without correction for multiple testing. Statistical maps were analyzed with Xjview [25], which allowed us to identify the brain regions associated with the detected clusters.

To compare the general characteristics of the two groups, we used a χ^2^ test for qualitative data and a Student’s *t*-test for quantitative data. We used a Kruskal-Wallis test to compare the three subgroups according to the type of hallucination. A difference was considered significant at *P* < 0.05.

## Results

The DLB-hallu and DLB-c groups were comparable with regard to age, sex and MMSE score (see Table 1). In terms of clinical symptoms, there was no significant difference between the two groups for the primary criteria for DLB. However, fluctuations seemed to be more frequent in the DLB-hallu group, but this result did not reach statistical significance (*P* = 0.07, 95% confidence interval: 0.059, 1.2351).

**Table 1:** **Clinical and therapeutic characteristics of the DLB-hallu and the DLB-c groups (** ***N*** **= 66)** ^**a**^

		**DLB-hallu (** ***n*** **= 36)**	**DLB-c (** ***n*** **= 30)**	***P*** **-value**
Mean age, yr (SD)		71.7 (10.2)	73.5 (6.9)	0.41
Sex (M/F)		19/14	18/10	0.89
Mean MMSE score (SD)		21.7 (5.6)	23.3 (4.3)	0.20
Extrapyramidal syndrome (score^b^)				
Akinesia (0/1/2/3/4)		3/18/11/4/0	4/19/5/2/0	0.12
Rigidity (0/1/2/3/4)		4/25/5/2/0	5/15/7/3/0	0.469
Tremor at rest (0/1/2/3/4)		29/7/0/0/0	22/7/0/0/0	0.733
Fluctuations		32	21	0.07
History of depression		13	12	0.74
Delirium episode		7	5	0.77
Sleep disturbance		12	9	0.74
Neuroleptics		13	2	0.006
Dopaminergic medication		4	8	0.10
AChEI		16	9	0.22

^a^AChEI, Acetylcholinesterase inhibitor; DLB-c, Control group of patients with dementia with Lewy bodies who were not having visual hallucinations; DLB-hallu, Study group of patients with dementia with Lewy bodies who were having visual hallucinations; MMSE: Mini Mental State Examination; SD, Standard. Data are number of patients, unless otherwise indicated.

With regard to cognitive performance, there were no significant differences between the two groups (all *P* > 0.05) (see Table 2). The DLB-c group performed worse than the DLB-hallu group on the FCSRT, and the DLB-hallu group performed worse than the DLB-c group on the TMT A, but without a significant difference.

**Table 2:** **Comparison of cognitive ability between the DLB-hallu and DLB-c groups (** ***N*** **= 66)** ^**a**^

		**DLB-hallu (** ***n*** **= 36)**		**DLB-c (** ***n*** **= 30)**		***P*** **-value**
		**Tested patients,** ***n*** **(%)**	**Mean (SD)**	**Tested patients,** ***n*** **(%)**	**Mean (SD)**	
FAB (18^b^)		21 (58)	10.7 (4.49)	19 (63)	10.7 (4.37)	0.967
FCSRT						
IR (16^b^)		22 (61)	14 (1.72)	15 (60)	13.8 (3.14)	0.637
FR (48^b^)		22 (61)	16.7 (7.53)	15 (60)	13.2 (10.11)	0.276
TR (48^b^)		22 (61)	39.6 (8.46)	15 (60)	32.7 (14.37)	0.160
CFR (16^b^)		20 (56)	6.2 (3.48)	13 (43)	5.3 (3.73)	0.592
CTR (16^b^)		20 (56)	13.8 (2.73)	13 (43)	12.9 (3.39)	0.353
TMT A^c^		17 (47)	99.5 (51.08)	21 (70)	92.9 (47.34)	0.713
TMT B^c^		17 (47)^d^	176.2 (89.42)	21 (70)^e^	215.1 (104.07)	0.339
Digit span test						
Direct		20 (56)	4.8 (1.28)	16 (53)	4.6 (0.89)	0.555
Indirect		19 (53)	3.2 (1.23)	16 (53)	2.8 (0.86)	0.358
Lexical evocation						
Formal		21 (58)	10.4 (7.70)	17 (57)	10.7 (7.32)	0.596
Semantic		23 (64)	16.2 (9.96)	17 (57)	14.9 (7.89)	0.784
Rey-Osterrieth Complex Figure Test (36^b^)		11 (31)	29 (6.4)	13 (43)	28.7 (8.87)	0.704

^a^CFR, Cued free recall; CTR, Cued total recall; DLB-c, Control group of patients with dementia with Lewy bodies who were not having visual hallucinations; DLB-hallu, Study group of patients with dementia with Lewy bodies who were having visual hallucinations; FAB, Frontal assessment battery; FCSRT, Free and Cued Selective Remaining Test; FR, Free recall; IR, Immediate recall; SD, Standard; TMT, Trail Making Test; TR, Total recall. ^b^Total possible score. ^c^Measured in seconds. ^d^Seven patients failed to finish this test. ^e^Nine patients failed to finish this test.

The DLB-hallu group was more often treated with neuroleptics: seven by clozapine, two by olanzapine, two by tiapride, one by cyamemazine and one by aripiprazole.

Within the DLB-hallu group, we divided patients into three subgroups according to the type of hallucination. Group 1 (*n* = 8) included patients with visual illusions (for example, feeling of movement or deformation of an object). Group 2 (*n* = 9) consisted of patients with simple hallucinations with vision of an isolated entity (for example, a person or an animal). Group 3 (*n* = 6) comprised patients who had complex hallucinations with the vision of scenes (for example, several persons). For 13 patients, we had insufficient information on the type of hallucinations they were having. We did not find any significant difference between the three subgroups according to the type of hallucination on the basis of clinical data (age, fluctuations, extrapyramidal syndrome) or on the basis of neuropsychological tests, except for direct digit span test (4 in the group 2 versus 5.75 in the group 3, *P* = 0.033).

### Cerebral perfusion

#### DLB-hallu versus DLB-c

**Figure 1:**
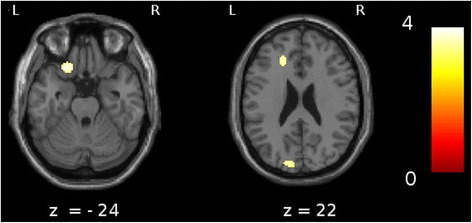
**Comparison of patients with dementia with Lewy bodies with versus without hallucinations.** A comparison of patients with dementia with Lewy bodies (DLB) with versus without hallucinations (*P* < 0.001, including age and type of radiotracer as nuisance covariates and a minimum cluster size of 25 voxels) revealed significant hypoperfusion in the left anterior cingulate gyrus (Brodmann area (BA) 32), the left orbitofrontal cortex (BA 11/47) and the left cuneus (BA 19).

Analyses revealed significant hypoperfusion (*P* < 0.001) in three brain regions in the DLB-hallu group compared with the DLB-c group: the left anterior cingulate cortex (ACC) within the limbic regions (Brodmann area (BA) 32), the left orbitofrontal cortex (BA 11 and 47) and the left cuneus within the occipital cortex (BA 18) (Figure 1 and Table 3). To evaluate the influence of neuroleptics on these results, we performed supplementary analyses comparing DLB-hallu group and DLB-c group after exclusion of patients who were taking neuroleptics. The results were similar to those of the previous analysis, with additional significant hypoperfusion in the left fusiform gyrus (*P* < 0.001) in the DLB-hallu group compared with the DLB-c group.

**Table 3:** **Brain regions of relative hypoperfusion associated with presence of hallucinations or with severity of hallucinations** ^**a**^

**Brain regions**		**BA vicinity**	**Side**	**Voxels,** ***n***	***t*** **-value**	**Coordinates MNI (mm)**			***P*** **-value**
						***x***	***y***	***z***	
Presence of hallucinations									
	Anterior cingulate cortex	32	L	111	3.73	−18	34	22	<0.001
	Orbitofrontal cortex	11/47	L	307	4.15	−20	26	−28	<0.001
	Cuneus	18	L	118	3.59	−12	−88	24	<0.001
Severity of hallucinations									
	Anterior cingulate cortex	32	L	557	3.77	−18	8	40	<0.001
		32	R	557	3.89	10	12	44	<0.001
	Orbitofrontal cortex	11/47	L	493	4.29	−22	24	−30	<0.001
	Parahippocampal gyrus	20	R	791	4.40	40	−20	−20	<0.001
	Cuneus	18	L	185	3.78	−16	−88	20	<0.001

^a^B, Bilateral; BA, Brodmann area; L, Left; MNI, Montreal Neurological Institute space; R, Right. Minimum cluster size = 25 voxels.

#### Severity of hallucinations

**Figure 2:**
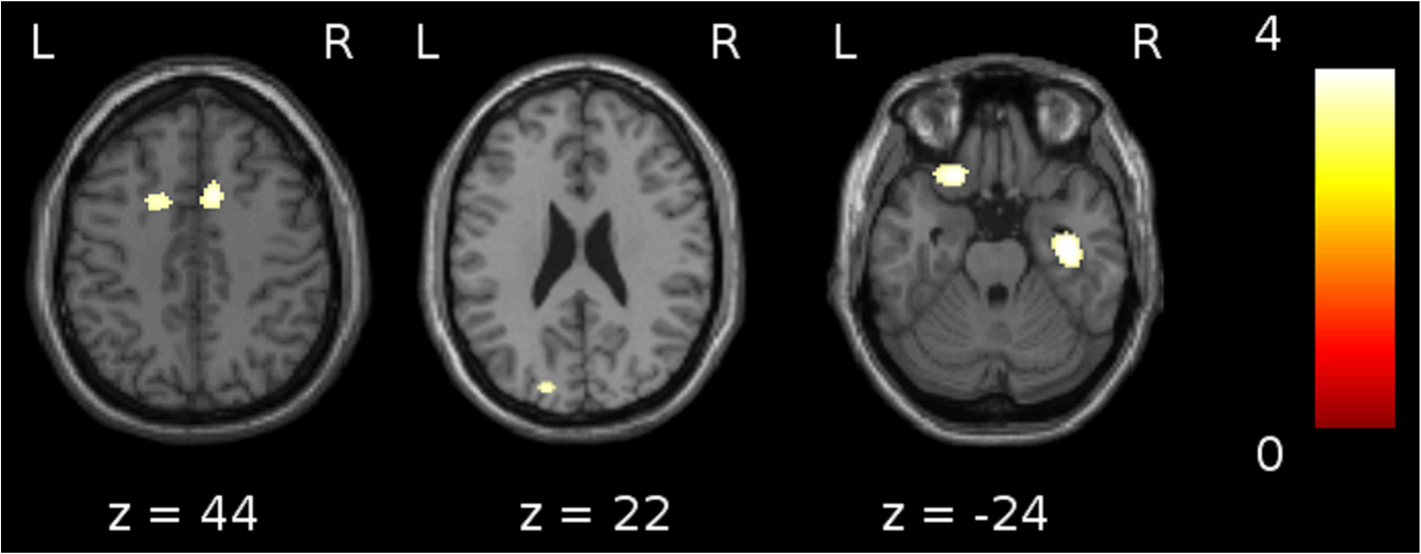
**Correlation analyses between cerebral hypoperfusion and severity of hallucinations in patients with dementia with Lewy bodies.** Correlation analyses between cerebral hypoperfusion and the severity of the hallucinations in the patients with dementia with Lewy bodies revealed the involvement of the bilateral anterior cingulate cortex (Brodmann area (BA) 32), the left orbitofrontal cortex (BA 11/47), the right parahippocampal gyrus (BA 20) and the left cuneus (BA 18) (*P* < 0.001, including age and type of radiotracer as nuisance covariates and a minimum cluster size of 25 voxels).

In correlation analysis of cerebral perfusion regarding the severity of hallucinations in the patients with DLB, we found significant hypoperfusion in the bilateral ACC (BA 32), the right parahippocampal gyrus, the right inferior temporal gyrus (BA 20), the left orbitofrontal cortex (BA 11 and 47) and the left cuneus (BA 18) (Figure 2 and Table 3).

## Discussion

In this work, we studied two groups of patients with DLB, one with and the other without VHs, who were broadly comparable for general and clinical characteristics. We found three regions with hypoperfusion in patients with DLB and VH. The first was posterior and consisted of the occipital cortex (cuneus), which is involved in visual information processing. The second corresponded to the ACC, which is involved in control process and error detection. The third corresponded to the orbitofrontal cortex, which is involved in inhibitory control and has a network lateralized primarily to the left.

In our study, hypoperfusion in the cuneus (BA 18) seemed to be associated with the presence of hallucinations. This secondary visual area, BA 18, is involved in the recognition and extraction of object features (shape, color, position in space, movement). Dysfunction of BA 18 causes errors in visual processing, with occurrence of object distortions that explain visual illusions particularly well. Patients with DLB report visual illusions more frequently than true hallucinations [26]. The localized relative dysfunction of this visual area, which we found in the present study, is consistent with previous data reported in the literature [9,10,12,15,27]. It confirms the original hypothesis that the occipital dysfunction specific to DLB [28,29] might be involved in the occurrence of VHs. The implication of visual areas was previously confirmed by other studies [10,12,16], in association with the parietal cortex [10] or the right temporo-occipital junction [12]. In previous work by our group involving patients with AD chosen from the Alzheimer’s Disease Neuroimaging Initiative database, we showed atrophy of the occipital cortex in patients with VHs. In our study, the left occipital region seemed predominant. Nagahama *et al*. [10] also found a predominance of left occipital involvement. This lateralization suggests that impairment of visual perception is crucial in VHs, for which the dominant pathway is the left occipitotemporal cortex, whereas visuospatial function preferentially follows the right occipitoparietal pathway [30,31].

In addition to these posterior aspects, we found hypoperfusion in the left ACC (BA 32) and the left orbitofrontal cortex (BA 11 and 47), suggesting that involvement of the occipital cortex alone is not sufficient to cause hallucinations. The ACC is a part of the limbic lobe, which is activated in tasks involving attention on the Stroop test and go/no-go tasks and is supposed to play an important role in attention, motivation, executive function and error detection [32-34]. The ACC, aside from the insula, is also known to contain the neurons called von Economo neurons [35], which would be implicated in intuitive decision making. Thus, in a complex situation where a quick decision is needed, these cells would be crucial to detect errors and make the correct decision. The functional deficit of the ACC in patients with DLB could lead to difficulty in error detection and correct decision making. The orbitofrontal cortex is well known for this role in inhibitory control and decision making [36]. Dysfunction of this area could prevent the patient from inhibiting the production of internal images.

The following hypothesis on the occurrence of hallucinations in DLB can therefore be proposed. Secondary visual areas are deficient and send false data to the entire cortex (ascending or bottom-up phenomenon). The patient cannot recognize this information as abnormal, because the ACC and the orbitofrontal cortex are also impaired and the VH seems real (descending or top-down phenomenon). The presence of hallucinations requires both a lesion of visual areas and one of control regions such as the ACC and orbitofrontal cortex. Previous studies have implicated the cingulate gyrus in the genesis of hallucinations [37]. It has been found to be the case for the ACC in patients with AD and for the posterior cingulate cortex in patients with DLB [10,11]. Menthis *et al*. found a significant hypometabolism in orbitofrontal and cingulate areas bilaterally in patients with AD who had delusional misidentification syndromes [38].

As in the study by Nagahama *et al*. [10], the left cingulum was implicated in our study, whereas the right hemisphere has been observed to be more particularly affected in most studies. Nevertheless, we noted bilateral involvement of the ACC when we took the severity of the hallucinations into account. Severity was defined here not by the frequency of hallucinations, but by their type. A hallucination was considered mild if it was an illusion and severe if it was a complex scene. On the basis of our results, we suggest that the severity of VH depends on the extent to which the system of error detection within the ACC is impaired, with unilateral dysfunction being sufficient for visual illusions, whereas bilateral involvement would be necessary for the vision of complex and aberrant scenes. The severity of hallucinations also correlated with the inferior temporal cortex and parahippocampal hypoperfusion. These results are consistent with previously published data. Harding *et al*. [39] found an association between hallucinations and high densities of Lewy bodies in the parahippocampal and inferior temporal cortices. In a recent study, Megevand *et al*. [40] showed that VHs could be evoked by direct electrical stimulation of the parahippocampal area. Our results support the role of the parahippocampal gyrus in the perception of visual scenes [41] because its hypoperfusion seems necessary for the occurrence of complex VHs, but not for visual illusions.

Our study has some limitations. The diagnosis of DLB was based on clinical features, and we did not have access to the postmortem examinations to confirm these diagnoses. However, the McKeith criteria for DLB have very good specificity (98%) [42]. Exploration of hallucinations is difficult because they are transient and short, and SPECT is often performed outside the hallucinatory period. Functional imaging studies to show which areas are involved during hallucinations would be interesting, but they are difficult to achieve because hallucinations are not predictable and require considerable cooperation on the part of the patient, which is not easy to obtain in patients with DLB. A higher proportion of patients with hallucinations were on neuroleptics, and these drugs could have influenced the SPECT findings. Handley *et al*. showed frontal hypoperfusion secondary to neuroleptics (haloperidol and aripiprazole) in healthy volunteers, but anterior cingulate perfusion was increased after neuroleptic treatment [43]. The same results were found by Pardo *et al*. [44]. The relative hypoperfusion found in the ACC is possibly minimized by neuroleptics.

## Conclusions

Overall, our study, together with previous studies, suggests that the occurrence of VHs in DLB requires the dysfunction of both the anterior and posterior regions, which are involved in top-down and bottom-up mechanisms, respectively. VHs seem to be related to impairment of secondary visual areas involved in visual perception and impairment of the ACC and orbitofrontal cortex involved in control processes and error detection. Well-formed hallucinations with complex scenes seem to be related specifically to impairment of bilateral ACC and parahippocampal gyrus involved in the perception of visual scenes.

## Abbreviations

ACC: Anterior cingulate cortex
AChEI: Acetylcholinesterase inhibitor
AD: Alzheimer disease
BA: Brodmann area
CFR: Cued free recall
CTR: Cued total recall
DLB: Dementia with Lewy bodies
DLB-c: Control group of patients with dementia with Lewy bodies who were not having visual hallucinations
DLB-hallu: Study group of patients with dementia with Lewy bodies who were having visual hallucinations
FAB: Frontal Assessment Battery
FCSRT: Free and Cued Selective Reminding Test
FR: Free recall
IR: Immediate recall
MMSE: Mini Mental State Examination
MNI: Montreal Neurological Institute
MRI: Magnetic resonance imaging
SPECT: Single-photon emission computed tomography
SPM: Statistical Parametric Mapping
TMT: Trail Making Test
TR: Total recall
VH: Visual hallucination
